# Co-mutations of *CTNNB1* and *PTEN* drive aggressive tumor progression in endometrial cancer

**DOI:** 10.1242/dmm.052788

**Published:** 2026-06-18

**Authors:** Shamsun Nahar, Eunhee M. Jeong, Keun Cheon Kim, Russell Broaddus, Jung-Yoon Yoo, Jae-Wook Jeong, Kyeong A. So, Tae Hoon Kim

**Affiliations:** ^1^ Department of Obstetrics, Gynecology and Women's Health, University of Missouri School of Medicine, Columbia, MO 65203, USA; ^2^ Department of Pathology and Laboratory Medicine, University of North Carolina, School of Medicine, Chapel Hill, NC 27599, USA; ^3^ Department of Biomedical Laboratory Science, Yonsei University Mirae Campus, Wonju 26493, Republic of Korea

**Keywords:** Endometrial cancer, β-catenin (CTNNB1), PTEN depletion, Epithelial-mesenchymal transition (EMT)

## Abstract

Endometrioid endometrial cancer (EEC) is the most prevalent gynecological malignancy. While early-stage EEC typically carries a favorable prognosis, late-stage EEC shows significantly poorer outcomes, with a five-year survival rate of only 17−19%. Co-occurring *PTEN* and *CTNNB1* variants are frequent in EEC and linked to poor prognosis. To investigate their functional impact, we generated uterine-specific *Pten* knockout mice and dominantly stabilized *Ctnnb1* mutant mice (*Pgr^cre/+^Pten^f/f^Ctnnb1^f(ex3)/+^*; *Pten^d/d^Ctnnb1^f(ex3)/+^*). These double-mutant mice exhibited significantly reduced survival compared to single *Pten* knockout (*Pgr^cre/+^Pten^f/f^*; *Pten^d/d^*) mice. Histopathology revealed aggressive, metastatic endometrioid cancer in *Pten^d/d^Ctnnb1^f(ex3)/+^* mice; these mice developed myometrial invasion by four weeks of age – unlike *Pten^d/d^* mice. Transcriptomic analysis identified activation of multiple oncogenic pathways, including WNT/CTNNB1, PI3K/AKT, basal cell carcinoma, sonic hedgehog signaling and epithelial−mesenchymal transition (EMT). Immunohistochemistry confirmed hallmark features of EMT in the uterus of double-mutant mice, including strong downregulation of E-cadherin (CDH1) and upregulation of the EMT regulator SNAIL (Snai1). These findings demonstrate that synergistic mutations of PTEN and CTNNB1 promote early invasion, EMT activation, and metastatic progression, offering mechanistic insight into the aggressive behavior and poor clinical outcomes associated with this subtype of EEC.

## Teaser

**Summary:** PTEN loss and activating CTNNB1 mutations synergistically drive aggressive endometrial cancer by promoting early invasion, EMT activation, metastasis, and significantly reduced survival.

## INTRODUCTION

Endometrial cancer is the most common malignancy of the female reproductive system and the fourth most common cancer among women in the USA, with an estimated 69,120 new cases and 13,860 deaths projected in 2025 (National Cancer Institute. Introduction to the endometrial SPOREs. 2025). Notably, it is the only major cancer for which survival has declined over the past four decades (Siegel et al., 2025). Its incidence increases with age and is associated with several physiological risk factors, including obesity, diabetes and hypertension (Gu et al., 2021; Yin et al., 2014). Histologically, ∼90% of newly diagnosed cases are classified as endometrioid cancer (Dedes et al., 2011). Histologic subtypes include endometrioid (87.2%), serous (5.6%), carcinosarcoma (3.9%), clear cell (1.7%) and mixed type (1.7%) cancer (Watkins et al., 2021). Endometrioid cancer is graded from 1 to 3, whereas serous, clear cell and carcinosarcomas are considered high-grade histologies and have not been assigned International Federation of Gynecology and Obstetrics (FIGO) grades (Zheng, 2023). Non-endometrioid endometrial cancer is, by definition, high grade and not graded by using the FIGO system. Approximately 90% of newly diagnosed cases are classified as endometrioid cancer (Guo et al., 2025). Although early-stage disease is generally managed effectively with surgery, advanced or recurrent endometrial cancer can be challenging to treat, particularly in cases that are not mismatch repair (MMR)-deficient (MMRd) (Polidano et al., 2025). While blockade of immune checkpoint inhibitors, including that of programmed cell death 1 (PDCD1, also known as and hereafter referred to as PD-1), have demonstrated significant clinical benefit in MMRd tumors, resistance to conventional chemotherapy remains a concern in many patients (Eskander et al., 2023; Mirza et al., 2023). Combination approaches, such as chemotherapy plus PD-1 inhibitors, have shown improved outcomes, highlighting the importance of molecular profiling to guide personalized treatment strategies (Crosbie et al., 2022; Gordhandas et al., 2023).

Genomic profiling has revealed distinct molecular subtypes with prognostic significance beyond traditional clinicopathologic factors. The Cancer Genome Atlas (TCGA) classifies endometrial cancer into four molecular subgroups: (1) ultramutated, characterized by mutations of DNA polymerase epsilon catalytic subunit A (POLE) and excellent prognosis; (2) hypermutated, characterized by mismatch repair (MMR) deficiency and microsatellite instability (MSI); (3) copy number low [also known as microsatellite stable (MSS)],with relatively low overall mutation burden and; (4) copy number high, typically serous-like tumors with extensive copy-number alterations, frequent mutations of TP53 and associated with poor outcomes (Bell and Ellenson, 2019). Beyond these subtypes, frequent mutations in *PTEN*, *CTNNB1*, PIK3CA, *ARID1A* and *KRAS* have been identified in endometrial cancer (Bell and Ellenson, 2019). Despite these insights, the mechanisms driving aggressive progression remain incompletely understood and are thought to involve complex interactions between loss of tumor suppressor and activation of oncogenic signaling pathways.

Two common alterations in endometrial cancer are mutations in the tumor suppressor gene phosphatase and tensin homolog (*PTEN*) and the oncogene catenin beta 1 (*CTNNB1*) (Brooks et al., 2019; Cancer Genome Atlas Research et al., 2013). *PTEN* encodes a lipid phosphatase that negatively regulates the PI3K/AKT/mTOR pathway by dephosphorylating phosphoinositide (3,4,5)-trisphosphate (PIP_3_) (Downes et al., 2007). *PTEN* mutations are detected in ∼25% of endometrial hyperplasia and ≤80% of endometrioid endometrial cancer (Mutter et al., 2000). Recent meta-analyses have reported that ∼33% of individuals diagnosed with atypical endometrial hyperplasia have concurrent endometrial cancer (Doherty et al., 2020). In the same study, the annual risk of progression to cancer was estimated to be 8.2% for those with untreated atypical endometrial hyperplasia and 2.6% for individuals with non-atypical endometrial hyperplasia (Doherty et al., 2020). Among the four TCGA molecular subtypes of endometrial cancer – ultramutated, hypermutated, copy-number low and copy-number high – *PTEN* mutations occur in 94%, 88%, 77% and 15% of cases, respectively (Cancer Genome Atlas Research et al., 2013). Somatic *PTEN* mutations of endometrial cancer are considered early events in tumorigenesis (Bell and Ellenson, 2019; Cheung et al., 2011; Levine et al., 1998; Nero et al., 2019). These mutations are primarily associated with endometrioid histology, younger age (<60), low-grade and early-stage disease, low recurrence rate and favorable clinical outcome (Watanabe et al., 2021). Consistent with this, uterine-specific *Pten* knockout mouse models are widely used to study the development of EEC (Daikoku et al., 2008; Kim et al., 2025). CTNNB1 is a critical component of the Wnt signaling pathway that regulates cellular proliferation, migration and survival (Bell and Ellenson, 2019). Approximately 25–30% of endometrioid tumors harbor mutation in exon 3 of *CTNNB1* (Travaglino et al., 2022). These mutations prevent *CTNNB1* degradation, result in stabilization and accumulation of CTNNB1, leading to transcriptional activation of WNT/CTNNB1 target genes that promote cell proliferation and survival (Parrish et al., 2022). *CTNNB1* mutations arise early in tumorigenesis of endometrial cancer, during the transition from endometrial atypical hyperplasia to cancer (Saegusa et al., 2001) and have been associated with poor outcomes in patients with low-risk endometrioid tumors (Kurnit et al., 2017; Liu et al., 2014; Myers et al., 2014). Clinically, *CTNNB1* mutations are enriched in the no specific molecular profile (NSMP) molecular subtype and associated with an increased risk of recurrence – even in patients with early-stage, low-grade disease – suggesting its potential role as a prognostic biomarker (Travaglino et al., 2022).

The hedgehog (HH) signaling pathway, particularly sonic hedgehog (SHH), plays a critical role in embryonic development and tissue patterning (Lum and Beachy, 2004). Aberrant SHH activation has been implicated in tumor initiation and progression across multiple cancer types (Hooper and Scott, 2005) and may contribute to early events in endometrial carcinogenesis (Liao et al., 2009). SHH signaling has been linked to regulation of epithelial–mesenchymal transition (EMT) and cellular plasticity (Ke et al., 2015; Kotulak-Chrzaszcz et al., 2021; Li et al., 2006; Xu et al., 2014). However, its interaction with PTEN loss and CTNNB1 activation in endometrial cancer remains poorly understood.

We investigated the cooperative effects of *PTEN* loss and *CTNNB1* exon 3 mutation in endometrial cancer by using genetically engineered mouse models. Our double-mutant mice (Pgr^cre/+^Pten^f/f^Ctnnb1^f(ex3)/+^; Pten^d/d^Ctnnb1^f(ex3)/+^) demonstrated synergistic effects, including enhanced *Ctnnb1* activation, reduced survival and aggressive metastatic tumor behavior compared to mice carrying a single *Pten* mutation. Transcriptomic and histological analyses revealed early activation of basal cell carcinoma signaling, including upregulation of SHH, which coincided with EMT induction as evidenced by E-cadherin (CDH1) reduction and SNAIL (SNAI1) overexpression in endometrial tumors of double-mutant mice. These findings suggest that the convergence of Wnt/CTNNB1 and SHH signaling pathways drives EMT and underlies the aggressive phenotype of endometrial tumors harboring these co-mutations, providing mechanistic insights and potential therapeutic targets for high-risk disease.

## RESULTS

### *Pten* and *Ctnnb1* co-mutations shorten survival and accelerate tumorigenesis in endometrial cancer

Although *PTEN* and *CTNNB1* represent two key genetic drivers in endometrial cancer (Konopka et al., 2007; Kurnit et al., 2017), the effect of their co-mutations has not been evaluated. To investigate the effect of *PTEN* and *CTNNB1* co-mutations on tumorigenesis of endometrial cancer, *Pten* floxed (*Pten^f/f^*) (Lesche et al., 2002) and *Ctnnb1* exon 3 floxed (*Ctnnb1^f(Ex3)/+^*) mice (Harada et al., 1999; Jeong et al., 2009) were bred to the *Pgr^cre^* mouse model (Soyal et al., 2005) to generate double mutation of *Pten* and *Ctnnb1* exon 3 in the uterus. We validated the CTNNB1 activation by using immunohistochemistry and compared its protein expression with that of single-knockout *Pten^d/d^* mice. The analysis revealed a significant increase (*P*<0.001), with 3-fold elevation of the cytoplasmic CTNNB1 expressions in the uterus of double-mutant mice compared with that in *Pten^d/d^* mice at 1 month of age (Fig. S1A). Notably, 27.26±4.84% of epithelial cells in the uterus of double-mutant mice exhibited nuclear CTNNB1 accumulation (Fig. S1B), which confirmed the activation of CTNNB1 (Jeong et al., 2009). In contrast, nuclear CTNNB1 was detected in only 0.40±0.37% of epithelial cells in the uterus of *Pten^d/d^* mice (Fig. S1B).

**Fig. 1.**
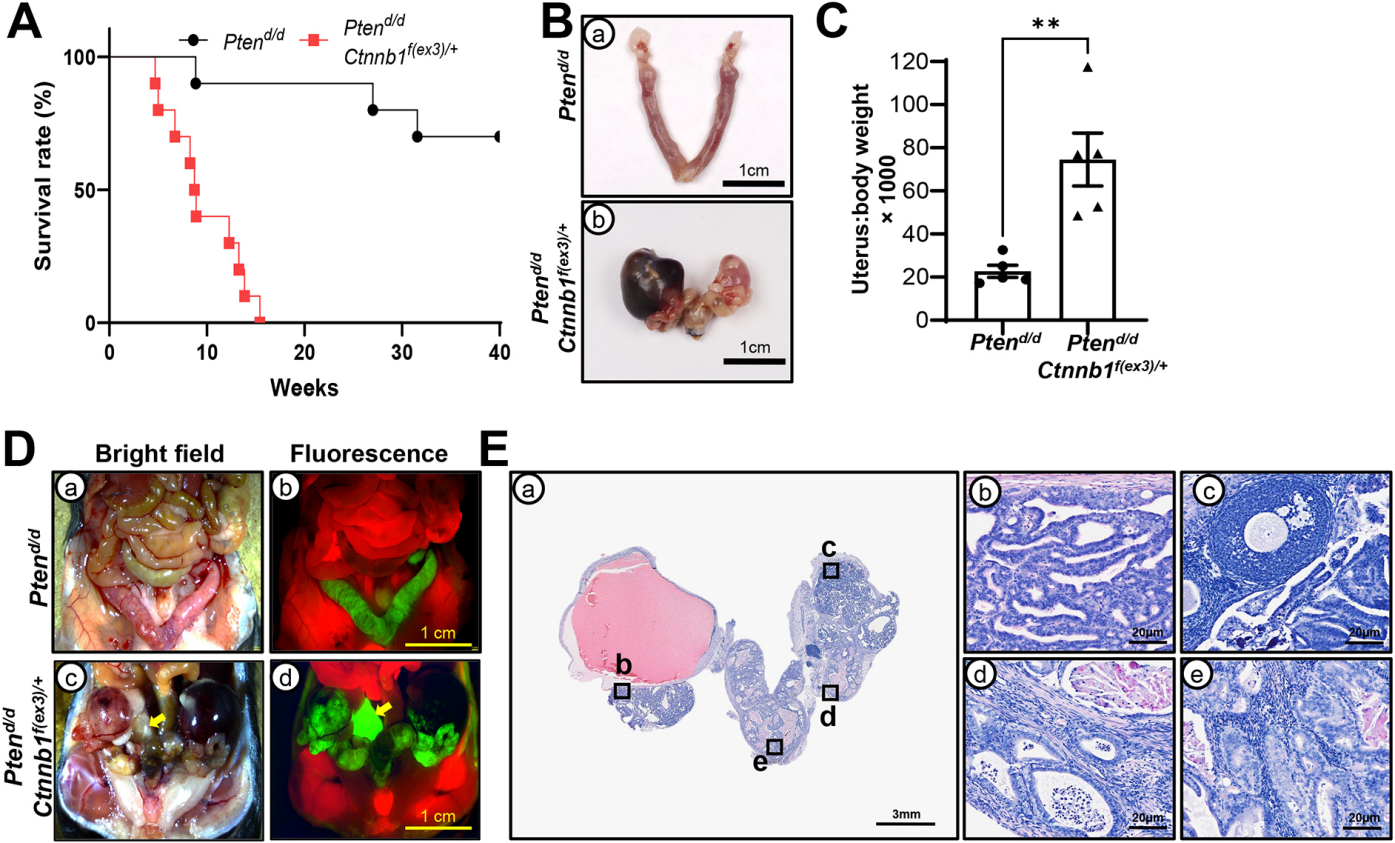
**Co-mutation of *Pten* and *Ctnnb1* shortens survival and accelerates tumorigenesis in endometrial cancer.** (A) Kaplan–Meier estimation plot to analyze survival of *Pten^d/d^* and *Pten^d/d^Ctnnb1^f(Ex3)/+^* mice over 40 weeks (*n=*10 per group). Plotted is the death due to endometrial cancer over time. Black dots indicate *Pten^d/d^* mice, red squares indicate *Pten^d/d^Ctnnb1^f(Ex3)/+^* mice. (B) Anatomical presentation of the uterus from *Pten^d/d^* (a) and *Pten^d/d^Ctnnb1^f(Ex3)/+^* (b) mice at 3 months of age. (C) Uterus-to-body weight ratios of *Pten^d/d^* (•) and *Pten^d/d^Ctnnb1^f(Ex3)/+^* (▴) mice (*n=*5). Pairwise comparisons were performed using Student's *t*-test with five biological replicates. Data are presented as mean±s.e.m.; **P<0.01. (D) Fluorescence images of the uterus from mice as indicated, using reporter (mT/mG) imaging to trace metastasis outside the uterus (a,c). GFP-positive uterine tissues and migrated cells are shown in green, other Td Tomato-positive tissues are shown in red (b,d). Yellow arrows indicate tumor formation in the peritoneal cavity (bright green) of *Pten^d/d^Ctnnb1^f(Ex3)/+^* mice. (E) Representative image showing the histopathological examination of the whole uterus obtained from *Pten^d/d^Ctnnb1^f(Ex3)/+^*. Boxed areas are shown magnified on the right, showing tumor formation outside of the ovaries (b-c), metastasis to the ovaries (d) and involvement of the cervical region (e).

Next, we examined the lifespan of *Pten^d/d^* and *Pten^d/d^Ctnnb1^f(ex3)/+^* mice (*n=*10 per group) to assess the impact of CTNNB1 activation in the context of *PTEN* mutation by adapting the Kaplan–Meier estimator to analyze survival (Goel et al., 2010; Kim et al., 2014). Survival rates were significantly reduced in double-mutant mice compared with *Pten^d/d^* mice (*P*<0.001) (Fig. 1A). All *Pten^d/d^Ctnnb1^f(ex3)/+^* mice (100% penetrance) reached their humane endpoint due to progressive endometrial tumor burden by 16 weeks of age (median survival 8.8 weeks). In contrast, only four out of ten *Pten^d/d^* mice reached endpoint by 40 weeks, and median survival was not reached during the observation period. To examine the impact of *Pten* and *Ctnnb1* co-mutations on tumorigenesis of endometrial cancer, we evaluated gross morphology, tumor weight, and histological feature in *Pten^d/d^* and *Pten^d/d^Ctnnb1^f(ex3)/+^* mice at three months of age. Gross morphology showed that co-mutation of *Pten* and *Ctnnb1* led to more aggressive tumors spread beyond the uterus compared to single *Pten* mutation (Fig. 1B). Although overall body weight did not differ significantly between *Pten^d/d^* and *Pten^d/d^Ctnnb1^f(ex3)/+^* mice, uterus-to-body weight ratios were significantly (3.24-fold) increased in *Pten^d/d^Ctnnb1^f(ex3)/+^* mice compared to *Pten^d/d^* mice (74.52±12.27 mg versus 22.68±2.80 mg; *P*<0.01) (Fig. 1C).

Uncovering tumorigenesis mechanisms by using mouse models requires that metastatic cancer can be easily distinguished from surrounding normal tissues. Fluorescent imaging provides a sensitive and rapid method for cellular and molecular visualization *in vivo*. To enable this, we incorporated the *Rosa26^mTmG^* reporter into our endometrial cancer models, enabling GFP labeling of *Pgr-Cre* recombined uterine cells (Muzumdar et al., 2007). The fluorescent imaging revealed that *Pten^d/d^* mice developed primary endometrial tumors without GFP-positive cells outside the uterus (Fig. 1D). In contrast, *Pten^d/d^Ctnnb1^f(ex3)/+^* mice developed large primary endometrial tumors accompanied by GFP-positive lesions in the ovaries, cervix, and peritoneal cavity (Fig. 1D), consistent with metastatic dissemination of uterine-derived tumor cells rather than primary tumors in these organs. Histopathological analysis confirmed endometrial cancer with widespread neoplastic glands invading the myometrium (Fig. 1E), and detailed examination of ovaries and cervix verified the presence of tumor cells originating from the uterus (Fig. 1Eb–Ee).

### *Pten^d/d^Ctnnb1^f(ex3)/+^* mice develop aggressive and metastatic endometrial cancer

**Fig. 2.**
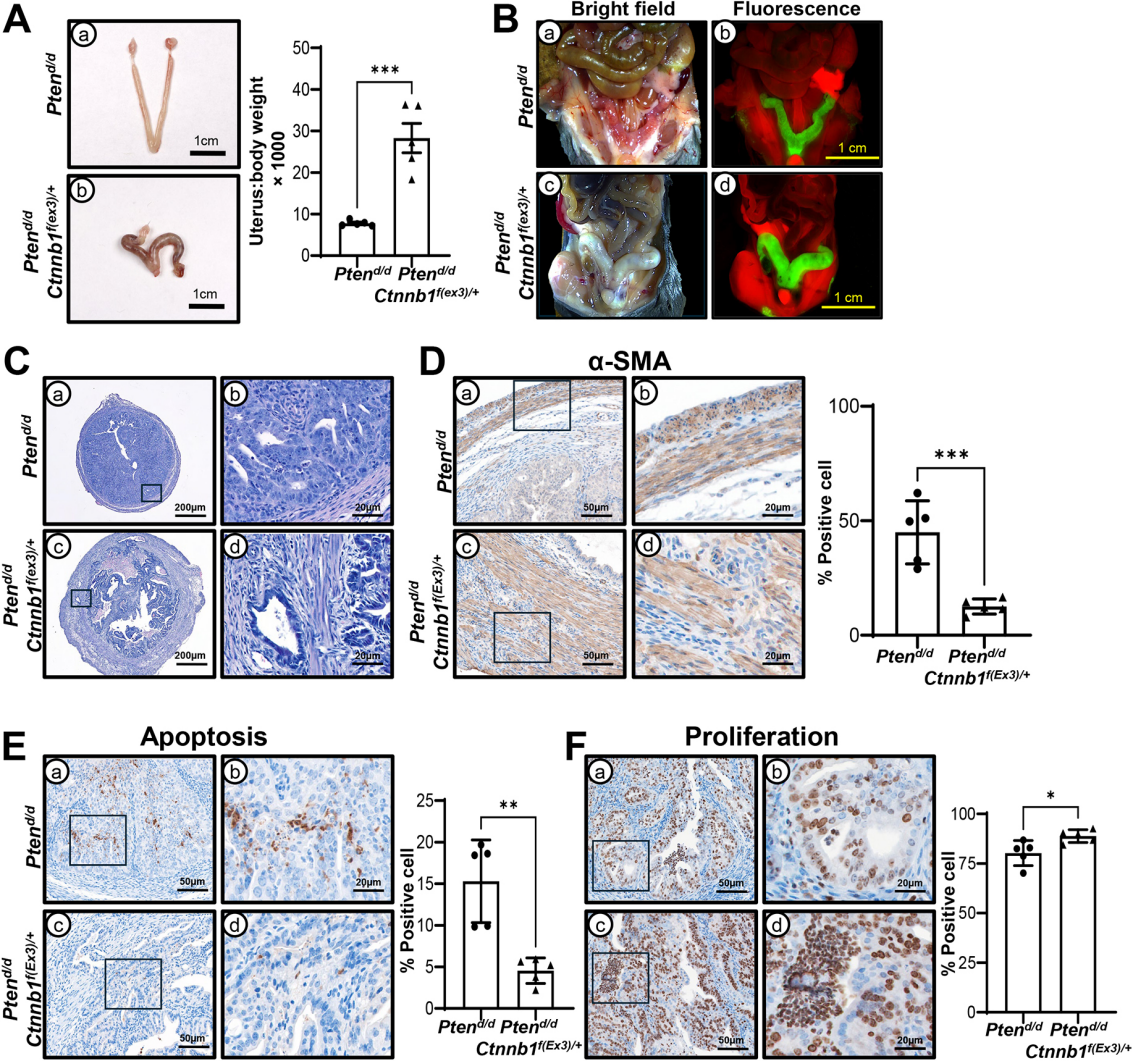
**Early emergence of invasive phenotypes in *Pten^d/d^* and *Pten^d/d^Ctnnb1^f(Ex3)/+^* mice at one month of age.** (A) Representative images of uterine morphology and uterus-to-body weight ratios of *Pten^d/d^* and *Pten^d/d^Ctnnb1^f(Ex3)/+^* mice at 1 month of age (*n=*5). ***P<0.001. (B) Fluorescent reporter (mT/mG) imaging of the body cavity of *Pten^d/d^* and *Pten^d/d^Ctnnb1^f(Ex3)/+^* mice to trace any uterus abnormalities of single- (a,b) and double-mutant (c,d) mice. In both types of mice, body cavities and organs stained positive for Td (tomato red), whereas both uteri stained positive for GFP (green). (C) Hematoxylin and eosin staining of a cross-sectioned uterus from *Pten^d/d^* (a,b) and *Pten^d/d^Ctnnb1^f(Ex3)/+^* (c,d) mice by. (D) Representative immunohistochemistry (IHC) images showing staining against smooth muscle actin (α-SMA) demonstrating any dysregulation and disruption of normal myometrial architecture in the uterus of single-mutant (a,b) and double-mutant (c,d) mice (*n=*5). Semi-quantitative analysis of the myometrial area was performed using Visiopharm software and presented as percentage of positive cells. (E) Representative images of the uterus in  *Pten^d/d^* (a,b) and *Pten^d/d^Ctnnb1^f(Ex3)/+^* (c,d) mice, immunostained for the apoptosis marker cleaved caspase-3 (*n=*5). Semi-quantitative analysis was done using Visiopharm software. (F) Representative images of the uterus in *Pten^d/d^* (a,b) and *Pten^d/d^Ctnnb1^f(Ex3)/+^* (c,d) mice, stained for the cell proliferation marker Ki67 (*n=*5). Semi-quantitative analysis was conducted as described in E. Pairwise comparisons were assessed by Student's *t*-test. Graphs were plotted using PraphPad Prism software. *P<0.05; **P<0.01; ***P<0.001.

To determine when invasive phenotypes begin to emerge, we carried out pathohistological analysis of mice aged 1 month. The uterus and ovaries of *Pten^d/d^* mice showed endometrial hyperplasia morphology (Fig. 2Aa). However, morphology of the uterus in *Pten^d/d^Ctnnb1^f(ex3)/+^* mice was severely deformed showing signs of inflammation and necrosis (Fig. 2Ab) along with a significantly increased uterus-to-body weight ratio compared to *Pten^d/d^* mice (28.29±3.52 vs 7.78±0.29, respectively; *P*<0.001) (Fig. 2A). The fluorescent images also showed that the uterus and ovaries of double-mutant mice were deformed and inflamed with the sign of necrosis (Fig. 2Bc,d). In contrast, *Pten^d/d^* mice exhibited only mild inflammation of uterus and showed normal ovaries (Fig. 2Ba,b). Importantly, tumor-cell invasion into the myometrium was observed in the double mutants but was not observed in single-mutant counterparts (Fig. 2C). To further characterize the neoplastic changes, expression of smooth muscle actin alpha 2 (ACTA2; hereafter referred to as α-SMA) and apoptosis marker cleaved caspase 3 were analyzed by using immunohistochemistry. The analysis revealed severe dysregulation of α-SMA, with the disruption of the normal myometrial cell arrangement (Fig. 2D). The percentage of α-SMA-positive cells was significantly reduced in the double mutant (12.54±1.32; *P*<0.001) compared to the single mutant (44.94±5.50). Furthermore, apoptosis (assessed by staining against the apoptosis marker cleaved caspase-3) was reduced more than 3-fold in the uterus of double-mutant *Pten^d/d^Ctnnb1^f(ex3)/+^* mice (4.54±0.69) compared to that of single *Pten^d/d^* mice (15.29±2.23) (Fig. 2E). In contrast, cell proliferation (assessed by staining against the cell proliferation marker Ki67) was significantly higher in double-mutant mice compared to single-mutant mice (88.81±1.45 vs 80.23±2.83; *P*<0.05) (Fig. 2F).

### Endometrial hyperplasia in *Pten^d/d^Ctnnb1^f(ex3)/+^* mice reveals reduced apoptosis and abnormal myometrial structure

**Fig. 3.**
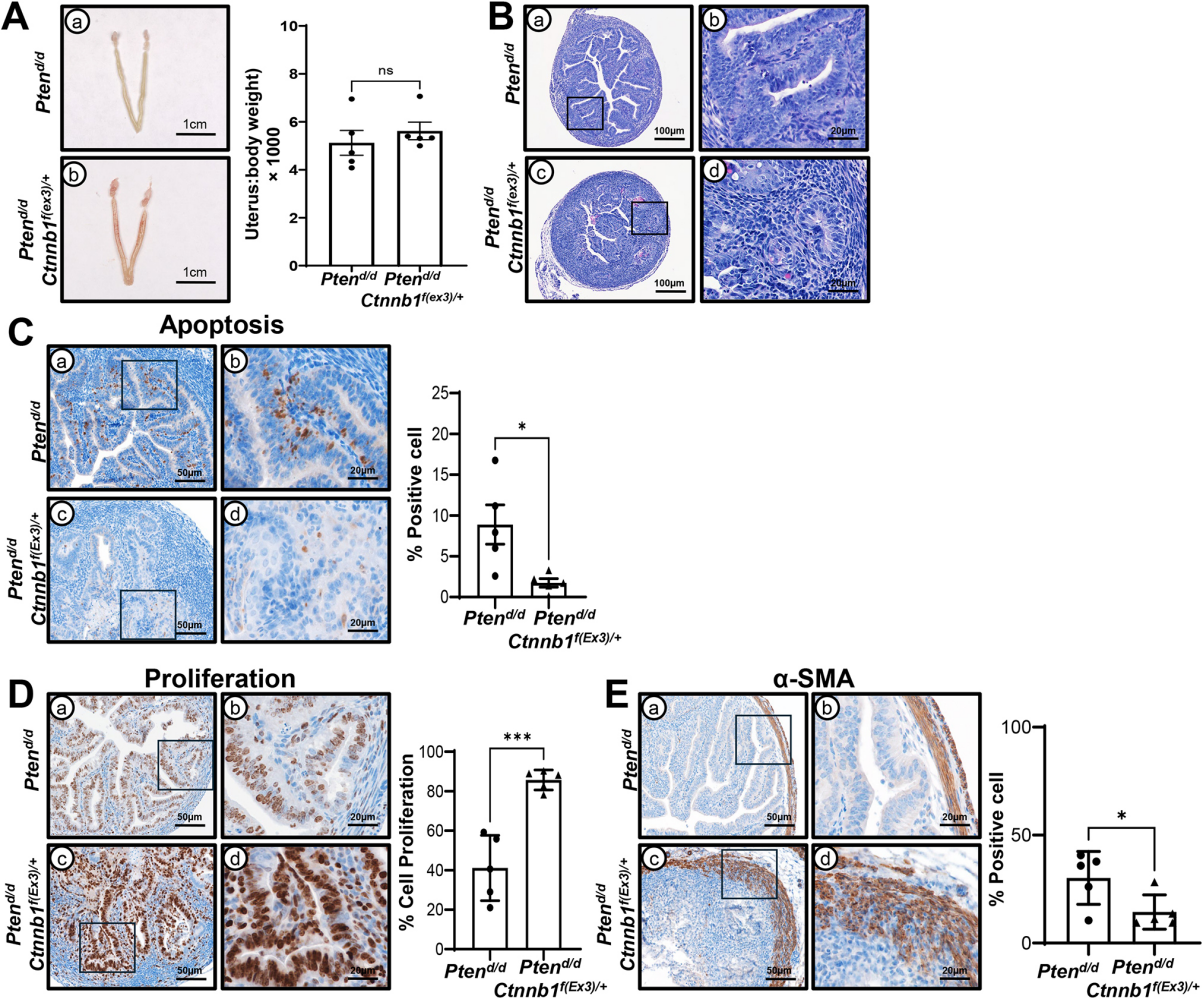
**Early molecular alterations in *Pten^d/d^Ctnnb1^f(Ex3)/+^* double-mutant mice at 2 weeks of age.** (A) Representative images of the uterine morphology (left) and uterus-to-body weight ratio (right) in *Pten^d/d^* and *Pten^d/d^Ctnnb1^f(Ex3)/+^* mice at two weeks of age (*n=*5). (B) Representative images of hematoxylin and eosin-stained uterus from *Pten^d/d^* and *Pten^d/d^Ctnnb1^f(Ex3)/+^* mice for histopathological examination (*n=*5). (C) Representative immunohistochemistry (IHC) images (left) and semi-quantitative analysis (right) of the uterus from *Pten^d/d^* (a,b) and *Pten^d/d^Ctnnb1^f(Ex3)/+^* (c,d) mice, stained for cleaved caspase-3 (*n=*5). (D) Representative IHC images (left) and semi-quantitative analysis (right) of the uterus from *Pten^d/d^* (a,b) and *Pten^d/d^Ctnnb1^f(Ex3)/+^* (c,d) mice, stained for Ki67 (*n=*5). Semi-quantitative analyses of the protein expressions were performed using AI-based image analysis software (Visiopharm) and presented as percentage (%). (E) Left: Representative IHC images of the uterus from *Pten^d/d^* (a,b) and *Pten^d/d^Ctnnb1^f(Ex3)/+^* (c,d) mice stained against smooth muscle actin (α-SMA), showing intact myometrium and disrupted myometrial structures (*n=*5). Right: Semi-quantitative analysis of the myometrial area was performed using Visiopharm software and is presented as the percentage of α-SMA-positive cells. Pairwise comparisons were assessed by Student's *t*-test. Graphs were plotted using PraphPad Prism software. ns, not significant (*P*>0.05); *P<0.05; ***P<0.001.

To better understand the molecular mechanisms underlying tumor initiation, we analyzed the uterus of 2-week-old mice before obvious differences in tumor burden emerged. At this age, both *Pten^d/d^* and *Pten^d/d^Ctnnb1^f(ex3)/+^* mice exhibited endometrial hyperplasia, and no significant difference in uterine-to-body weight ratio was observed between groups (*P*>0.05) (Fig. 3A). Histological evaluation confirmed hyperplasia in both groups without obvious structural abnormalities (Fig. 3B). However, apoptosis was significantly reduced in the uterus of double-mutant compared to single-mutant mice (1.74±0.51 vs 8.88±2.41, relatively; *P*<0.05) (Fig. 3C). Cell proliferation was significantly increased in the uterus of double-mutant compared to single-mutant mice (85.66±2.27 vs 41.10±7.39; *P*<0.001) (Fig. 3D). In addition, α-SMA protein expression was smooth and continuous in the uterus of *Pten^d/d^* mice (Fig. 3E), whereas that of double-mutant mice discontinuously expressed α-SMA within the myometrium (Fig. 3Dc,d). Semi-quantitative analysis also showed a significant reduction in α-SMA-positive cells in the double-mutant group (14.36±3.17; *P*<0.05) compared with the single-mutant group (30.16±4.91). These results indicate that, although *Pten^d/d^* and *Pten^d/d^Ctnnb1^f(ex3)/+^* mice both displayed endometrial hyperplasia, co-mutation of *Pten* and *Ctnnb1* uniquely suppressed apoptosis and disrupted myometrial integrity.

### Transcriptomic analysis identifies early oncogenic pathway activation

**Fig. 4.**
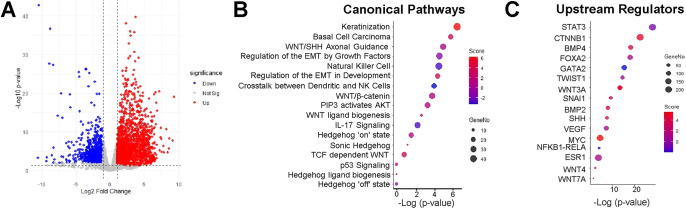
**Transcriptomic profiling reveals oncogenic pathway activation in *Pten^d/d^Ctnnb1^f(Ex3)/+^* mice at 2 weeks of age.** (A) Volcano plot illustrates 3427 differentially expressed genes (DEGs), including 2213 upregulated (red) and 1214 downregulated (blue) transcripts in the uterus of double-mutant mice (fold change >±2; false discovery rate <0.05). Non-significant transcripts are shown in gray. (B) Dot plot illustrates pathway dysregulation and activation of multiple oncogenic pathways in the uterus of double-mutant mice. (C) Dot plot of the upstream regulator analysis, highlighting activation of CTNNB1, WNT3A, MYC, and epithelial–mesenchymal transition (EMT)-associated regulators. Pathway analysis was performed using Ingenuity Pathway Analysis (IPA). Dot color represents *z-*scores (blue=low, red=high), and dot size reflects the number of genes associated with each pathway.

To identify the molecular effects of *Pten* and *Ctnnb1* co-mutations, we performed RNA sequencing (RNA-seq) analysis of the uterus from 2-week-old mice (*n=*4). Transcriptomic profiling identified 3427 differentially expressed genes (DEGs), including 2213 upregulated and 1214 downregulated transcripts in the uterus of double-mutant mice (fold change >±2; false discovery rate <0.05) (Fig. 4A). The QIAGEN Ingenuity Pathway Analysis (IPA) revealed activation of multiple oncogenic signaling pathways in the uterus of double-mutant mice (Fig. 4B). Notably, WNT/CTNNB1 and TCF-dependent WNT signaling were activated, consistent with the presence of stabilized CTNNB1, which is known to trigger oncogenic cell proliferation, survival, stemness (i.e. a high degree of plasticity) and differentiation (Zhang and Wang, 2020). Within WNT/CTNNB1- and TCF-dependent WNT signaling pathways, 32 and 23 DEGs from our dataset were identified, with activated *z-*scores of 0.76 and 3.55, respectively, and -log(p-values) of 3.93 and 0.81, respectively (Fig. 4B). The PIP_3_−AKT signaling axis was activated as well [27 DEGs; *z-*score: 1.35; -log(p-value): 3.46], reflecting a hallmark of proliferation and differentiation driven by loss of *Pten*. This differential activation likely reflects conditional deletion of *Pten* in these models, which promotes PIP_3_ accumulation and AKT activation. Although protein-level validation was not performed in this study, prior reports have demonstrated robust PI3K–AKT pathway activation following PTEN loss in endometrial cancer (see Kim et al., 2010, 2014). In addition, the basal cell carcinoma-signaling pathway was activated (*z-*score: 3.32; 21 upregulated molecules), largely regulated by HH signaling. Consistent with this, our transcriptomic data revealed an activated ‘Hedgehog-on’ state and inhibited ‘Hedgehog-off’ state, with upregulation with the SHH pathway (*z-*score: 2.24). Furthermore, regulation of EMT in development was significantly enriched [*z-*score: 3.3; -log(p-value): 4.54]. In contrast, immune-related pathways were suppressed, including natural killer (NK) cell signaling [*z-*score: −2.87; –log(p-value): 4.66] and crosstalk between dendritic and NK cells [*z-*score: −3.36; –log(p-value): 4.13] (Fig. 4B).

Upstream regulator analysis further identified CTNNB1 and WNT3A as significantly activated [*z-*scores: 4.66 and 4.75; -log(p-value): 22.58 and 13.47, respectively], confirming the activation and stabilization of canonical CTNNB1 signaling (Fig. 4C). A total of 252 DEGs were linked to these regulatory networks. The proliferation-associated transcription factor MYC was also strongly activated with 201 predicted target genes [*z-*score: 5.51; (-log(p-value): 4.55]. Importantly, several key EMT regulators, including BMP2, BMP4, SHH, TWIST1 and SNAI1, exhibited significant activation [-log(p-value)>8.00] with corresponding *z-*scores of 4.21, 3.65, 4.07, 0.67 and 3.08, respectively. Conversely, the upstream regulators GATA2 [*z-*score: −2.00, -log(p-value): 15.52] and NFKB1–RELA [*z-*score: −1.99, -log(p-value): 4.01] were significantly inhibited (Fig. 4C). Taken together, these transcriptomic data suggest that *Pten* and *Ctnnb1* co-mutations not only activate canonical oncogenic cascades, such as WNT/CTNNB1, PI3K/AKT and MYC*,* but also aberrantly engage the basal cell carcinoma signaling pathway through SHH activation.

### Aberrant SHH signaling in *Pten^d/d^Ctnnb1^f(ex3)/+^* mice

**Fig. 5.**
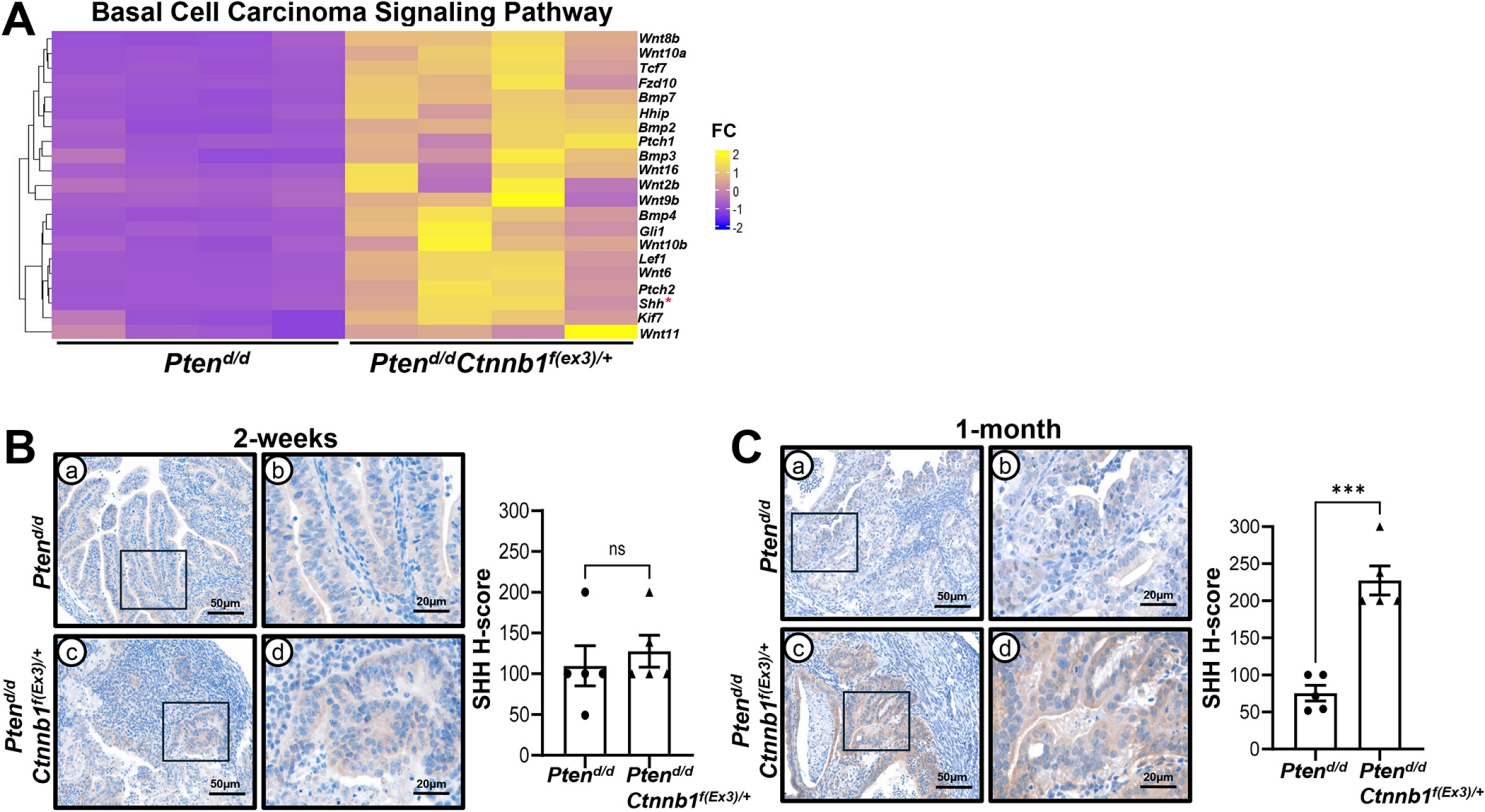
**Aberrant activation of SHH in *Pten^d/d^Ctnnb1^f(Ex3)/+^* mice.** (A) Heatmap illustrates the dysregulating activation of 21 DEGs in basal cell carcinoma signaling pathway. Activation and inhibition are color coded as yellow and blue, respectively. FC, fold change. (B) Representative immunohistochemistry (IHC) images and semi-quantitative analysis of the sonic hedgehog (SHH) protein in the uterus of *Pten^d/d^* (a,b) and *Pten^d/d^Ctnnb1^f(Ex3)/+^* (c,d) mice at 2 weeks of age (*n=*5). (C) Representative IHC images of SHH protein in the uterus of *Pten^d/d^* (a,b) and *Pten^d/d^Ctnnb1^f(Ex3)/+^* (c,d) x at 1 month of age (*n=*5). Pairwise comparisons were assessed by Student's *t*-test. Graphs were plotted using PraphPad Prism software. ns, not significant (*P*>0.05); ***P<0.001.

Basal cell carcinoma is driven primarily by constitutive activation of the HH signaling pathway which leads to aberrant activation of the HH signaling pathway and tumor growth, often reinforced by crosstalk with other oncogenic pathways (Rubin et al., 2005). Therefore, we examined the basal cell carcinoma signaling pathway. Consistent with IPA findings, canonical pathway mapping revealed significant upregulation [-log(p-value): 5.97] of 21 DEGs related to basal cell carcinoma, including members of the *Bmp*, *Wnt*, *Ptch*, *Hedgehog* gene families, and *Tcf7*, *Fzd10*, *Lef1* and others (Fig. 5A). Notably, the genes encoding hedgehog-interacting protein (HHIP) and SHH were among the most significantly upregulated in the uterus of *Pten^d/d^Ctnnb1^f(ex3)/+^* mice (fold change = 17.22 and 58.02, respectively; *P*<0.05 for both) (Fig. 5A), supporting the predicted activation of the HH signaling pathway (Fig. 4B).

*Shh* is the key regulator of the SHH signaling pathway and has been increasingly recognized as a critical driver of cancer development and progression (Cong et al., 2025). To validate the transcriptomic prediction of aberrant SHH signaling pathway activation, we examined SHH protein expression in the uterus of *Pten^d/d^* and *Pten^d/d^Ctnnb1^f(ex3)/+^* mice at 2 weeks and 1 month of age (*n=*5). At 2 weeks, no significant difference was observed between the groups (*P*>0.05) (Fig. 5B). However, by 1 month, SHH expression was significantly elevated in the uterus of *Pten^d/d^Ctnnb1^f(ex3)/+^* compared to *Pten^d/d^* mice (227±19.54 vs 75.37±10.61, respectively; *P*<0.001) (Fig. 5C). These findings demonstrate that aberrant activation of the SHH signaling pathway is a prominent feature in the uterus of *Pten^d/d^Ctnnb1^f(ex3)/+^* mice, emerging early, intensifying with age and contributing to a pro-tumorigenic environment.

### EMT is activated in endometrial tumors of *Pten^d/d^Ctnnb1^f(ex3)/+^* mice

**Fig. 6.**
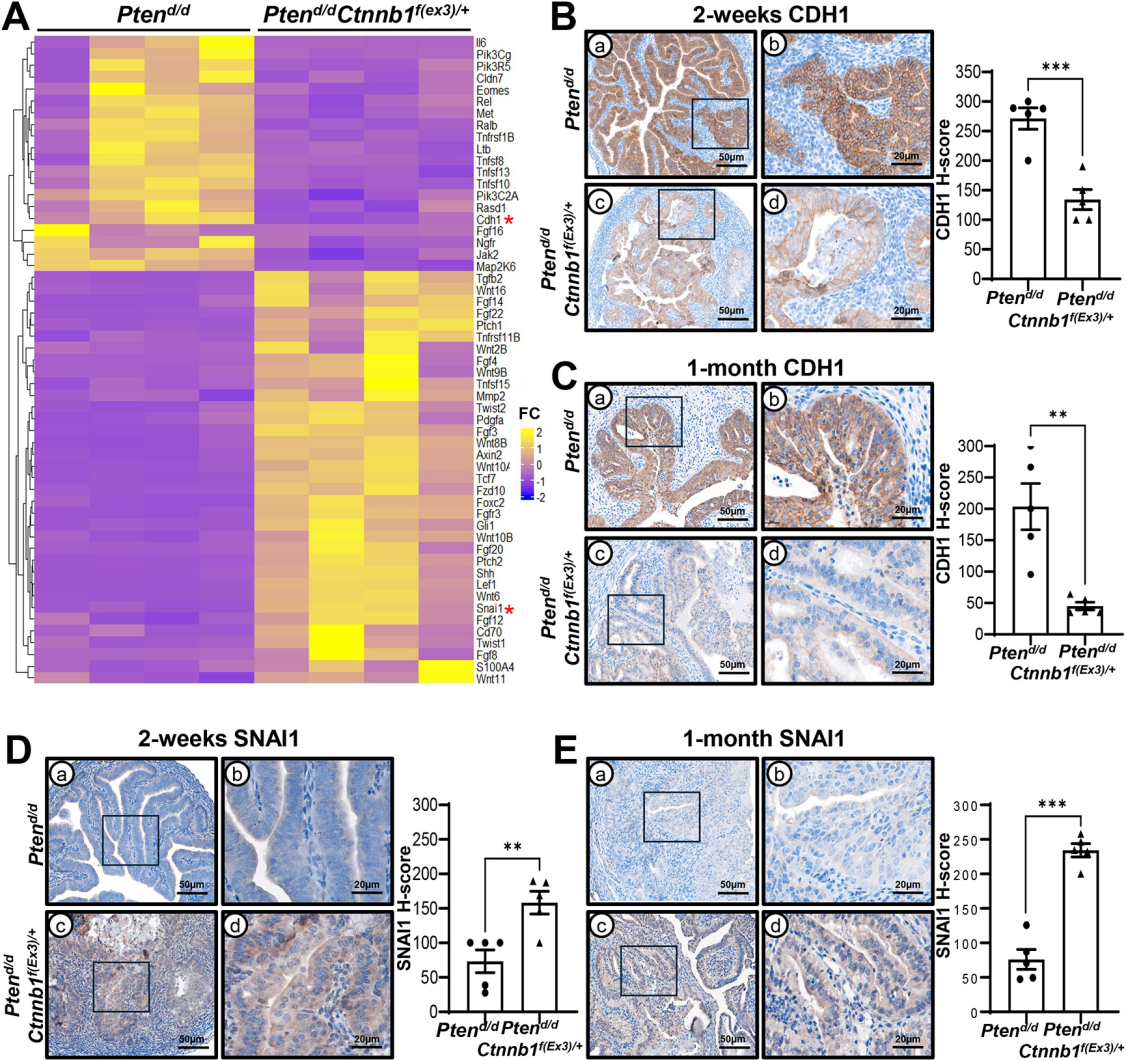
**Aberrant SHH and CTNNB1 signaling promotes EMT in *Pten^d/d^Ctnnb1^f(Ex3)/+^* mice.** (A) Heatmap demonstrates 58 DEGs in the EMT-regulatory pathways. Activated genes are shown in yellow, inhibited genes in blue. Red asterisks indicate downregulation of E-cadherin (*Cdh1*) and upregulation of *Snai1*. FC, fold change. (B) Representative immunohistochemistry (IHC) images and semi-quantification of the CDH1 expression in the uterus of *Pten^d/d^* (a,b) and *Pten^d/d^Ctnnb1^f(Ex3)/+^* (c,d) mice at 2 weeks of age (*n=*5). (C) Representative IHC images and semi-quantification of CDH1 expression in the uterus of *Pten^d/d^* (a,b) and *Pten^d/d^Ctnnb1^f(Ex3)/+^* (c,d) mice at 1 month of age (*n=*5). (D) Representative IHC images and semi-quantification of SNAI1 expression in the uterus of *Pten^d/d^* (a,b) and *Pten^d/d^Ctnnb1^f(Ex3)/+^* (c,d) mice at 2 weeks of age (*n=*5). (E) Representative IHC images and semi-quantification of SNAI1 expression in the uterus of *Pten^d/d^* (a,b) and *Pten^d/d^Ctnnb1^f(Ex3)/+^* (c,d) mice at 1 month of age (*n=*5). Semi-quantification of the cytoplasmic expression was performed using AI-based image analysis software (Visiopharm) at both time points. Pairwise comparisons were assessed by Student's *t*-test. Graphs were plotted using GraphPad Prism software. Data are presented as mean±s.e.m. **P<0.01; ***P<0.001.

The SHH pathway has been reported to be a key upstream regulator of EMT in multiple cancer progression (Berrino and Omar, 2024; Guen et al., 2017). Given its established role in driving cellular plasticity, we investigated whether aberrant activation of SHH signaling is associated with EMT induction in endometrial cancer from *Pten^d/d^Ctnnb1^f(ex3)/+^* mice. Consistent with pathway analysis indicating EMT activation, we validated key EMT markers. The transcriptomic profiling showed dysregulation of 58 EMT-regulating genes in *Pten^d/d^Ctnnb1^f(ex3)/+^*, including downregulation of *Cdh1* and upregulation of *Snai1* (Fig. 6A). On the one hand, immunohistochemical studies confirmed these findings, showing 2-fold reduction (*P*<0.001) in CDH1 expression in the endometrium of double mutant (134.27±16.94) compared to single mutant mice (271.28±18.12) at 2 weeks of age (Fig. 6B). By 1 month, CDH1 expression in the uterus of *Pten^d/d^Ctnnb1^f(ex3)/+^* mice was further reduced, ∼4.5-fold relative to that in *Pten^d/d^* mice (45.01±6.17 vs 203.62±36.89; *P*<0.01) (Fig. 6C). On the other hand, SNAI1 expression was markedly increased in double mutants (Fig. 6D,E). At 2 weeks, SNAI1 expression was significantly higher in double mutants (158.26±16.55) than in single mutants (73.24±16.48; *P*<0.01) (Fig. 6D). By 1 month, SNAI1 expression was further elevated (*P*<0.001) in double mutants (234.21±9.71) compared to single mutants (76.00±14.38) (Fig. 6E). These results demonstrate that aberrant HH and CTNNB1 signaling actively promote EMT, which likely underlies the observed adenomyosis and myometrial invasion, providing mechanistic insight into the aggressive phenotype of these tumors.

## DISCUSSION

Our study demonstrates that co-mutations of *Pten* and *Ctnnb1* in the uterus markedly accelerate endometrial tumorigenesis, reduce survival and drive aggressive metastatic disease. Although *PTEN* and *CTNNB1* are individually established as drivers in endometrial cancer, their synergistic effects have not been comprehensively evaluated. CTNNB1 stabilization in double-mutant mice induced cytoskeletal remodeling and invasive phenotypes, with WNT/CTNNB1 signaling promoting EMT. Transcriptomic analyses revealed activation of basal cell carcinoma and SHH signaling, which cooperate with WNT/CTNNB1 activation to enhance EMT, characterized by CDH1 loss and SNAI1 upregulation. These findings highlight a convergence of developmental and oncogenic pathways in driving aggressive endometrial cancer progression.

Human genomic studies have similarly revealed a strong co-occurrence of *PTEN* and *CTNNB1* mutations in endometrioid endometrial cancer (Kurnit et al., 2017; Liu et al., 2014). A previous study of 56 endometrioid endometrial cancer found 77.8% of *CTNNB1*-mutant tumors also harbored *PTEN* alterations (Konopka et al., 2007). In a larger cohort of 82 EECs, 25.6% exhibited *CTNNB1* mutations and, notably, 90% of these tumors also carried *PTEN* mutations (Watanabe et al., 2021). Preclinical evidence further supports the functional synergy of these alterations. Epithelial-specific *PTEN* loss causes endometrial hyperplasia, whereas combined *CTNNB1* exon 3 mutation promotes invasive endometrial cancer with myometrial invasion, particularly under ovarian insufficiency (Terakawa et al., 2019). Consistently, our double-mutant mice demonstrated that the addition of oncogenic mutations of *CTNNB1* markedly enhances tumor aggressiveness, leading to more invasive endometrial cancer.

Mechanistically, nuclear accumulation of CTNNB1 was significantly elevated in the uterus of double-mutant mice, consistent with activation of canonical WNT/CTNNB1 signaling. This molecular alteration was associated with significantly reduced survival – *Pten^d/d^Ctnnb1^f(ex3)/+^* mice succumbed to endometrial cancer within 16 weeks, whereas mortality in single *Pten* mutants was delayed and less frequent. Comprehensive morphological, histological and fluorescent imaging analyses revealed that co-mutant mice develop highly aggressive tumors, characterized beyond the uterus by extensive invasion to the ovaries, cervix and surrounding tissues. This invasive phenotype was accompanied by profound cytoskeletal remodeling, disruption of myometrial integrity and suppression of apoptosis, all of which are likely to contribute to early tumor initiation and rapid progression. Notably, at early stages, the uterus of double-mutant mice exhibited reduced apoptosis and discontinuous α-SMA expression, indicating that impaired myometrial integrity and suppressed cell death contribute to aggressive phenotype. Collectively, these findings established *Pten^d/d^Ctnnb1^f(ex3)/+^* mice as a robust preclinical model for aggressive and metastatic endometrial cancer. Importantly, our preclinical observations are concordant with human data – as demonstrated by Kurnit et al. (2017) – which showed that *CTNNB1* mutations in low-grade, early-stage endometrioid endometrial carcinomas identify a subset of patients with significantly increased cancer recurrence risk. These findings establish CTNNB1 activation as a key determinant of aggressive tumor biology and further strengthen the translational relevance of our mouse model for endometrial cancer.

Transcriptomic analyses revealed early and sustained upregulation of WNT/CTNNB1 and basal cell carcinoma signaling, particularly the SHH pathway, which precedes obvious tumor formation (Terakawa et al., 2019). The Wnt/CTNNB1 pathway is highly conserved and regulates key processes in the female reproductive system, including development, proliferation, survival, adhesion, motility and menstrual cycle regulation (Fatima et al., 2021). Aberrant activation of this pathway promotes proliferation, survival and metastatic potential in multiple cancers (Anastas and Moon, 2013). In endometrial cancer, constitutive activation of canonical Wnt/CTNNB1 signaling occurs early in tumorigenesis, with up to 40% of cases (predominantly endometrioid) harboring pathway abnormalities (Fatima et al., 2021; Lax, 2004).

SHH is one of the three Hedgehog ligands and has been extensively studied for its important role in the development of cancer (Cong et al., 2025). HH signaling is a driver of cancer stemness, cellular plasticity and tumor progression (Berrino and Omar, 2024; Sari et al., 2018). Aberrant SHH upregulation precedes obvious tumor formation and intensifies with age, suggesting it contributes to establishing a pro-tumorigenic microenvironment (Berrino and Omar, 2024; Sari et al., 2018). Our analyses identified aberrant SHH activation as an early and prominent feature of double-mutant mice. SHH pathway activation coincided with enhanced CTNNB1 signaling and preceded overt tumor invasion, suggesting cooperative interactions between developmental and oncogenic pathways in driving aggressive tumor behavior. Transcriptomic enrichment of basal cell carcinoma-associated signaling, together with marked upregulation of SHH protein, supports functional activation of this pathway in compound mutant uteri. Notably, SHH activation was associated with loss of CDH1 and upregulated SNAI1, indicating promotion of EMT. Although SHH signaling may exhibits context-dependent roles in endometrial cancer, our findings suggest that, in the setting of concurrent *PTEN* loss and *CTNNB1* activation, SHH pathway activation contributed to EMT and invasive disease progression.

EMT is a fundamental biological process involved in embryonic development, wound healing, fibrosis and cancer metastasis (Lamouille et al., 2014; Oh et al., 2013; Yang and Weinberg, 2008; Yoo et al., 2020). It is regulated by several transcription factors – such as SNAI1, SLUG, TWIST, ZEB1 and SIP1 – that suppress epithelial markers, like CDH1, to promote mesenchymal characteristics (Peinado et al., 2007; Yoo et al., 2020). Transcriptomic and immunohistochemical analyses revealed marked downregulation of *Cdh1* and upregulation of *Snai1*, indicating that EMT is actively promoted by *Pten* and *Ctnnb1* co-mutations. We found a marked reduction of CDH1 expression alongside a significant increase in SNAI1-positive cells in the endometrium of double-mutant mice, indicating a transition from epithelial to mesenchymal phenotype. These EMT-associated changes likely enhance cellular plasticity, migration and invasiveness – features often linked to therapeutic resistance and poor prognosis in high-grade and recurrent endometrial cancers (Kalluri and Weinberg, 2009). SHH activation appears to cooperate with WNT/CTNNB1 signaling to promote EMT, thereby linking developmental signaling programs with oncogenic progression. The convergence of WNT/CTNNB1 and SHH signaling provides a mechanistic explanation for the aggressive phenotype observed in double-mutant mice, including myometrial invasion, and metastasis to the ovaries and cervix. Notably, nuclear CTNNB1 accumulation coincided with induction of EMT, as evidenced by CDH1 downregulation and SNAI1 upregulation. These findings support the role of WNT/CTNNB1 signaling as a key driver of EMT and invasive behavior in endometrial cancer. Spatial transcriptomics of tumors with nuclear and non-nuclear mutant *CTNNB1* revealed distinct transcriptional profiles based on protein localization (Parrish et al., 2025). Nuclear regions showed enrichment in Wnt signaling and EMT pathways, whereas non-nuclear regions were associated with hormone signaling (Parrish et al., 2025). These findings highlight the similarity between our mouse model and human disease, and support further investigation into the therapeutic potential of targeting Wnt and nuclear CTNNB1 signaling in EEC.

Despite the strengths of our genetically engineered model, limitations should be noted. First, while this model recapitulated key molecular and histopathological features of aggressive endometrial cancer, it did not fully capture the genetic heterogeneity and microenvironmental complexity of human tumors. Second, our study primarily focused on early tumor initiation and progression; longer-term analyses of metastatic colonization and therapy response were not performed. Future studies should investigate molecular alterations in double-mutant mice later-stage to better define factors that drive disease progression and to identify potential therapeutic targets.

In this study, we demonstrated that co mutations of *CTNNB1* and *PTEN* influenced EMT in endometrial cancer. While this approach allowed us to define early molecular responses to these mutations, additional characterization of lineage and differentiation markers, including PAX8, cytokeratins and hormone receptors, will be important in future studies to further establish the relevance of these findings to human disease. Because *Pgr-Cre* drives recombination in both uterine epithelial and stromal compartments, the observed phenotype likely reflects combined effects across multiple cell types as well as their interactions. This may result in a more pronounced phenotype compared with epithelial-restricted Cre driver, such as *Ltf-iCre* (Daikoku et al., 2014; Liang et al., 2018). Given that PTEN and CTNNB1 are expressed in both stromal and epithelial compartments, their effects on uterine function are mediated, at least in part, through epithelial–stromal crosstalk. Identifying the molecular mediators of this intercompartmental signaling will be critical for understanding how these pathways contribute to uterine homeostasis and disease progression. Nevertheless, a major strength of this study is the use of an *in vivo* genetically engineered mouse model that faithfully reproduces early and progressive features of aggressive endometrial cancer, providing mechanistic insights that are directly relevant to human disease. Addressing the above limitations in future studies will further strengthen the translational relevance of this model to understand endometrial cancer pathogenesis and therapeutic development.

In conclusion, this study demonstrated that *PTEN* loss and *CTNNB1* activation synergistically drive oncogenic reprogramming in the uterus, promoting EMT and accelerating tumor progression through convergence of WNT/CTNNB1 and SHH signaling. EMT emerged as a central mechanism underlying epithelial disruption, myometrial invasion and metastatic potential, while also contributing to tumor cell survival, resistance to apoptosis and structural remodeling of the uterus. These findings established *Pten/Ctnnb1* co-mutant mice as a robust preclinical model of aggressive endometrial cancer and revealed that SHH signaling, in cooperation with WNT/CTNNB1 activation, promotes EMT and invasive behavior. Our results not only provided mechanistic insights into the poor prognosis associated with *PTEN* and *CTNNB1* co-mutations but also identified SHH signaling as a promising therapeutic target in high-risk disease.

## MATERIALS AND METHODS

### Mice and tissue collection

All experimental procedures involving animals were conducted in compliance with institutional and regulatory guidelines. Animal studies received prior approval from the University of Missouri's Animal Care and Use Committee (ACUC), under protocol number 65323. Mice with a uterus-specific deletion of *Pten*, i.e. *Pgr^cre/+^Pten^f/f^Rosa26^mTmG/mTmG^* (referred to as *Pten^d/d^*) (Kim et al., 2010) and *Ctnnb1* activation at exon 3, i.e. *Pgr^cre/+^Ctnnb1^f(ex3)/+^Rosa26^mTmG/mTmG^*; *Ctnnb1^f(ex3)/+^* (Jeong et al., 2009) were bred to generate double-mutant mice with a *Pten* deletion and *Ctnnb1* activation at exon 3 (*Pgr^cre/+^Pten^f/f^Ctnnb1^f(ex3)/+^Rosa26^mTmG/mTmG^*; *Pten^d/d^Ctnnb1^f(ex3)/+^*). In Pgr^cre/+^Rosa26^mTmG/+^ mice, uterine cells that express the progesterone receptor (Pgr) are labeled with mG (green), whereas cells lacking Pgr expression are labeled with mT (red) (Figs 1D, 2B) (Yoo et al., 2022). The CTNNB1 activation was further confirmed by immunohistochemical analysis and compared the protein expression with single-knockout *Pten^d/d^*. Tumors from both mouse models were collected at specific time points, i.e. at 2 weeks, and at 1 and 3 months of age (*n=*5 per group). Uterine tissues were either immediately flash-frozen after dissection and stored at −80°C for RNA extraction or fixed in 4% (w/v) paraformaldehyde for subsequent histological examination (Kim et al., 2014).

### RNA-sequencing and pathways analysis

Following the methodology reported by Li et al. (2024), total RNA was isolated from whole uterine tissue obtained from *Pten^d/d^* and *Pten^d/d^Ctnnb1^f(ex3)/+^* mice aged 2 weeks (*n=*4 independent biological replicates per genotype), which served as the discovery cohort for differential gene expression and pathway analysis. RNA was extracted using the RNeasy Total RNA Isolation Kit (Qiagen, Cat. #74106). The RNA concentration and purity were initially assessed via UV spectrophotometry with a NanoDrop instrument. Prior to sequencing, RNA samples were evaluated for quality and quantified to ensure concentrations of 100–200 ng/μl with RNA integrity numbers (RIN) above 8.0, as verified by the University of Missouri Genomics Technology Core. Library preparation for RNA sequencing was performed using 500 ng of total RNA and the KAPA mRNA HyperPrep kit (Kapa Biosystems, Wilmington, MA, USA). RNA was fragmented to produce fragments ∼300–400 bp in length. Indexed adapters (TruSeq UD from Illumina, San Diego, CA, USA) were ligated to cDNA prior to PCR amplification. The quality and quantity of the prepared libraries were validated using the Agilent DNA High Sensitivity chip and Promega's QuantiFluor dsDNA System. Libraries were pooled and subjected to 50 bp paired-end sequencing using an Illumina NovaSeq6000 system (S2, 100 bp kit; RRID:SCR_016387), achieving a read depth of ∼50 million reads per sample.

Raw sequencing data underwent base calling using Illumina RTA3, followed by demultiplexing and FastQ conversion via Bcl2fastq v1.9.0 (RRID:SCR_015058). Low-quality sequences (average score<20) were excluded, and the remaining reads were aligned to the mm39 mouse genome using STAR 2.7.9a (RRID:SCR_004463). Expression levels were quantified as read counts. Differential gene expression analysis between *Pten^d/d^* and *Pten^d/d^Ctnnb1^f(ex3)/+^* groups was conducted using the EdgeR software (RRID:SCR_012802), applying thresholds of >50 maximum expression counts, fold change >± 2, and the false discovery rate <0.05. Enrichment of the canonical pathways and identification of the upstream regulators were assessed through Ingenuity Pathway Analysis (IPA; RRID:SCR_008653). Data visualization, including dot plots, heatmaps, volcano plot was created using the Tidyverse (RRID:SCR_019186), ComplexHeatmap (RRID:SCR_017270) and ggplot2 (RRID:SCR_014601) packages, respectively, using Rstudio (R-4.5.1; RRID:SCR_000432).

### Immunohistochemical and histological staining

Immunohistochemistry (IHC) was carried out using paraffin-fixed uterine tissues. After deparaffinization and rehydration, sections were blocked with 10% normal goat serum. Tissue sections were incubated overnight at 4°C with primary antibodies including anti-β-catenin (CTNNB1; Cell Signaling Technology, Cat# 8480, RRID:AB_11127855; 1:5000), anti-cleaved caspase-3 (Cell Signaling Technology, Cat# 9661, RRID:AB_2341188; 1:500), anti-alpha smooth muscle actin (Abcam, Cat# ab5694, RRID:AB_2223021; 1:1000), anti-SHH (Abcam, Cat# ab53281, 1:500), anti-CDH1 (Cell Signaling Technology, Cat# 3195, RRID:AB_2291471, 1:500), anti-Snai1 (SNAI1, Santa Cruz Biotechnology, Cat# sc-28199, RRID:AB_2239533, 1:500), and anti-Ki67 (Abcam, Cat# ab15580, RRID:AB_443209; 1:500). The following day, biotinylated goat anti-rabbit (Vector Laboratories, Cat# BA-1000, RRID:AB_2313606) or anti-mouse (Vector Laboratories, Cat# BA-9200, RRID:AB_2336171) secondary antibodies were applied, followed by incubation with horseradish peroxidase (Invitrogen, Cat#43-4323, 1:1000) for 1 h. Detection was achieved using the DAB substrate kit (Vector Laboratories, Cat# SK-4100, RRID:AB_2336382). Slides were counterstained with hematoxylin and lithium carbonate, followed by dehydration using an automated staining and mounting system (Gemini AS with ClearVue, Epredia, Kalamazoo, MI, USA). For histological analysis, paraffin-embedded sections were stained with hematoxylin and eosin using the same automated staining and mounting system.

### Image acquisition and statistical analysis

All stained slides were digitized using the Akoya PhenoImager HT scanner (RRID:SCR_023772) to maintain consistency and minimize observer bias. High-resolution images of tissue sections were created for later analysis by scanning them in bright-field mode with a 20× objective, which resulted in a resolution of 0.5 μm per pixel. Quantification of IHC signal intensity was performed using Visiopharm software (RRID:SCR_002285). DAB staining intensity was scored as follows: 0=negative, 1=weak, 2=moderate, 3=strong. Visiopharm's deep learning module was applied to identify both nuclear and cytoplasmic staining for each protein of interest, and to generate cell population data within the epithelium and stromal compartments. The H-score was calculated by measuring the percentage of cells with weak, moderate or strong staining by factors of 1, 2 or 3, respectively, yielding a final score of 0 to 300 (Budwit-Novotny et al., 1986). Pairwise comparisons were assessed by Student's *t*-test using at least five biological replicates. Data are presented as mean±s.e.m., with *P*<0.05 considered statistically significant.

## Supplementary Material

10.1242/dmm.052788_sup1 Supplementary information
