# Supplementary material for: Co-mutations of CTNNB1 and PTEN drive aggressive tumor progression in endometrial cancer
Source: Dis Model Mech. 2026 Jun 18;19(7):dmm052788. doi: 10.1242/dmm.052788 (PMC13312928; doi:10.1242/dmm.052788)
Supplement: Supplementary information [file dmm-19-052788-s1.pdf]

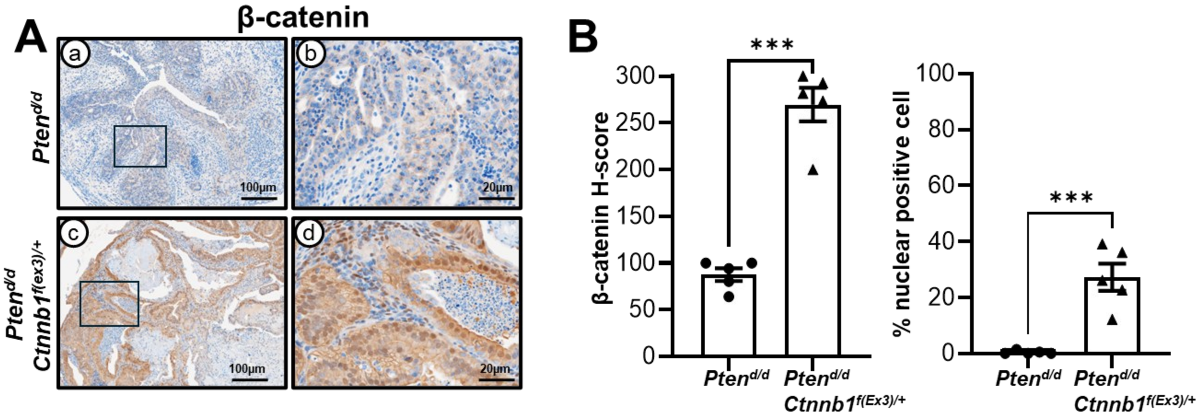

Fig. S1. Validation of β-catenin activation in Pten and Ctnnb1 double-mutant uteri. (A) Immunohistochemical analysis of β-catenin in uterine sections from *Pten*$^{d/d}$ and *Pten*$^{d/d}$ *Ctnnb1*$^{f(Ex3)/+}$ mice at 1 month of age. **(B)** Semi-quantitative assessment and H-scoring of cytoplasmic expression and nuclear accumulation of β-catenin in the same uterine sections and the scores were determined using the AI-based cell counting software Visiopharm. Pairwise comparisons were performed using Student's T-test with at least five biological replicates. Graphs were generated using GraphPad Prism. Data are presented as mean ± SEM. ***, $p < 0.001$.

Disease Models & Mechanisms • Supplementary information
